# Exploring the relationship between pain intensity and knee moments in participants with medial knee osteoarthritis: a cross-sectional study

**DOI:** 10.1186/s12891-021-04587-w

**Published:** 2021-08-12

**Authors:** Chen. Huang, Ping-Keung. Chan, Kwong-Yuen. Chiu, Chun-Hoi. Yan, Shun-Shing. Yeung, Siu N. Fu

**Affiliations:** 1 Department of Rehabilitation Sciences, The Hong Kong Polytechnic University, Hung Hom, Hong Kong, China; 2 Department of Orthopaedics and Traumatology, Queen Mary Hospital, The University of Hong Kong, Hong Kong, China; 3 Physiotherapy Department, MacLehose Medical Rehabilitation Centre, Hong Kong, China

**Keywords:** Knee osteoarthritis, External knee adduction moment, Pain

## Abstract

**Background:**

High biomechanical loading is believed to be a risk factor to pain in people with knee osteoarthritis (OA), but controversial findings have been reported on the relationship between external knee adduction moment (KAM) and pain. A more comprehensive analysis considering other factor such as external knee flexion moment (KFM) could help better reveal this relationship. This study explored the relationship between external knee adduction moment and pain intensity in participants with knee osteoarthritis (OA) using an integrated path analysis model.

**Methods:**

This was a cross-sectional study based on laboratory setting. Forty-seven participants with clinical and radiographic medial knee OA were analyzed for their external knee adduction moment (KAM) and knee flexion moment (KFM) during walking using a motion analysis system. Pain intensity was measured by visual analogue scale (VAS) and the pain subscale of the Knee Injury and Osteoarthritis Outcome Score. Varus/valgus alignment was captured and quantified using a bi-planar X-ray system. Using a path analysis model, the relationships between pain intensity, KAM, KFM, OA radiographic severity, knee varus angle and walking speed were examined.

**Results:**

The proposed path model met the goodness-of-fit criteria. Based on this model, KAM had a negative effect on VAS pain indirectly through the mediation of KFM. The model indicated KAM and KFM were negatively related to one another; and KFM was positively related to VAS. The KAM index, defined as (KAM/ (KAM + KFM)), was negatively related to VAS.

**Conclusions:**

Path analysis enabled the construction of a more integrated pathokinematic framework for people with knee OA. The KAM index which reflected the load sharing on the frontal and sagittal planes also revealed its relationship with pain. Re-distribution of mechanical loading from frontal to sagittal plane might be a strategy for pain avoidance associated with mechanical irritation.

## Background

Knee osteoarthritis (OA) is a common problem in the senior population worldwide [1] and activity-related pain is the most predominant disabling symptom of this condition [2]. More importantly, there is an upward trend in the prevalence of knee pain associated with OA [3]. In people over 55 years of age, about 10 % had mild-to-moderate knee pain, and between 2.0 and 4.8 % would suffer from severe pain and disability caused by OA [4, 5]. The pain and disability would lessen their willingness to participate in physical and social activities [6], and strongly affect their health-related quality of life [7].

Most pain in knee OA is activity triggered especially in weight-bearing situations [8, 9]. Extrinsic and intrinsic factors that increase joint mechanical loading lead to greater intensity of knee pain [10]. Physically demanding occupations, habitual and intense physical activities had strong relationship with knee pain and joint degeneration [11]; people who were over-weight had over two fold higher risk of knee OA and obesity was related to 24.6 % of new onset of knee pain [11]. Excessive knee loading during gait, in particular, the external knee adduction moment (KAM), has been proposed as an essential intrinsic factor for OA related pain. Amin et al. found that seniors with higher peak KAM were more likely to develop chronic knee pain within 3–4 years [12]. Nevertheless, studies in search of relationship between KAM and pain intensity reported inconsistent findings [13–16]. In participants with mild radiographic knee OA, peak KAM was significantly higher in the symptomatic than the asymptomatic groups [13, 16]. However, a negative relationship between peak KAM and pain intensity was reported by Henriksen et al. [15]. It was also found that greater KAM impulse was related to higher pain intensity in participants with moderate radiographic knee OA [14, 15], but this was associated with lower pain intensity in participants with severe radiographic knee OA [14]. It is essential to consider the degree of OA severity when exploring the relationship between KAM and pain intensity in view of the fact that such a relationship was very likely to be specific to radiographic severity,

Peak external knee flexion moment (KFM) reflected joint loading, and the load would trigger pain in participants with knee OA. In people with symptomatic mild knee OA, the KFM at early stance phase was lower than their asymptomatic counterparts [13], but people with higher KFM were more likely to develop pain after exercises [17]. In view that KAM and KFM occur nearly simultaneously with the first peak of medial joint contact force at about the initial 23 % of the total gait cycle [18], Simic et al. reported that increase in KFM was associated with a reduction in KAM with gait modification [19]. They also found that KAM would drop but KFM would rise with toe-in gait; whereas the opposite was observed with toe-out gait during the first half of stance phase in people with knee OA [19]. These findings suggested an inverse relationship existed between KAM and KFM [20]. In order to better understand the relationship between KAM and pain, an analysis on the simultaneous change between KAM and KFM when taking into considerations of factors such as joint alignment [21] and walking speed [22] might better explain the direct relationship between KAM and pain.

Load sharing among the three anatomical planes has emerged as one of the mechanical outcome considerations in participants with knee OA. Asay et al. reported a transition of KFM-dominated total joint loading to a KAM-dominated loading in the long term, and the percentage of KAM over the total joint moment appeared to be associated with radiographic OA progression at follow-ups over a period of 5 years [23]. The percentage of KAM in total joint moment was associated with the change in medial-to-lateral knee articular cartilage thickness ratio in an 8-year follow-up. Hence, the proportion of KAM was possibly one key biomechanical factor linking to joint structure destruction in the initiation and progression of knee OA. Besides, the KFM and KAM contributed to 73 % of variance of the total joint force [24]. The external knee moment on the horizontal plane accounted for less than 1 % of total external joint moment [23] which was relatively low during the stance phase, thus its influence had been less emphasized. Therefore we aimed to explore whether the KAM index, which was the percentage of KAM over the sum of KAM and KFM, was associated with pain intensity in participants with medial knee OA. A cross-sectional relationship between KAM index and OA-related pain would help to establish their causal relationship.

The main goal of this study was to investigate the relationships between KAM and pain intensity in people with mild-to-moderate medial knee OA by path analysis taking the effects of KFM, disease severity, joint alignment and walking speed into considerations. We also explored whether load sharing represented by KAM index would have a relationship with pain intensity. It was hypothesized that (1) pain intensity could be determined by early stance KAM directly and indirectly through KFM; (2) there would be a positive association between KAM index and pain intensity in participants with mild-to-moderate medial knee OA.

## Methods

### Study design

This was an observational cross-sectional study.

### Participants

Participants were recruited from orthopedic department of a local hospital. The inclusion criteria were: (1) age between 50 and 80 years; (2) had minimal knee pain of 2 on an 11-point visual analogy scale (VAS) during level walking and with most painful site located in the medial knee compartment; and (3) plain x-ray revealed more degenerative changes in the medial than lateral compartment. Participants were excluded if they had any of the following: (1) history of low back or lower limb injury within the past 12 months; (2) low back, pelvis, hip, ankle or foot pain of VAS 3 or above; (3) rheumatoid arthritis, (4) knee valgus more than 3° [25], (5) history of knee surgery; (6) history of intra-articular injection in the past 3 months; (7) any other muscular, joint or neurological condition influencing lower limb function; (8) unable to walk independently without external assistance; or (9) body mass index (BMI) > 36 kg/ m^2^ [14]. Study size was reached according to the availability of eligible participants at the study period. Mild knee OA was identified as grade 1 and 2 in Kellgren-Lawrence (KL) grading scale and moderate groups as grade 3 [15].

### Outcome measurement

#### Pain intensity

Pain intensity was measured by both VAS and the pain subscale of the Knee Injury and Osteoarthritis Outcome Score (KOOS). The maximal level of pain intensity during walking in the past week was measured by an 11-point VAS with “0” represented no pain and 10 represented the worst pain. It quantified pain intensity in particular during walking in particular. Pain subscale from KOOS was also used to assess the pain intensity more comprehensively in different functional conditions instead of just walking as measured with the VAS. For each subscale, a 0-100 score scale was used with “0” represented the most severe knee problem, while “100” indicated no problem; thus higher scores indicated less pain. Convincing evidence from meta-analysis suggested KOOS had adequate content validity, internal consistency and construct validity [26], and excellent test-retest reliability of translated version has also been reported [26]. Participants were required to complete the subscale without any assistance from the assessor.

#### Knee joint kinetics

Knee joint kinetics was measured by a motion analysis system comprising 8 cameras (MX T40, Vicon, Oxford, UK) and 2 floor-mounted force plates (Kistler Group, Winterthur, Switzerland). The frame rate for the kinetic data was 100 Hz. Lower limb anthropometric information including knee width, ankle width and lower limb length were recorded. Sixteen reflective skin markers were attached according to the standard lower limb Plug-In-Gait marker set. The skin markers included bilateral anterior superior iliac spine, posterior superior iliac spine, thigh, knee, tibia, ankle, toe and heel [14]. Participant stood unshod and an initial recording was made during standing for lower limb modeling purpose. Afterwards, participants walked unshod in their comfortable speed on an 8-meter footpath. Adequate practice trials were provided for the participants to acquaint themselves with the test. Each participant was recorded for at least five walking trials. The five trials would be accepted for analysis only if both lower limbs had clean foot strike from heel-strike to toe-off on the force plates [27].

Vicon Nexus software (Version 2.5, Oxford, UK) was used to estimate the external knee joint moments with Lower limb Plug-In-Gait model settings. The periods between toe-off and heel strike were identified as when the magnitude of the force plate was below 10 N. Half of the stance phase was marked and peak KAM and KFM were respectively defined as the maximum values of knee moment in the frontal and sagittal plane during the initial 50 % of stance phase. They were normalized to body weight and reported in Nm/kg. All kinetic data were estimated by the average of five successful trials. KAM index was calculated as (KAM/ KAM + KFM)*100 as derived from Asay et al. [28].

#### Knee varus/valgus angle

Knee varus/valgus angle was examined with a low-dose bi-planar X-ray imaging system (EOS imaging, Paris, France) [29]. Participants stood with legs 4 cm apart so as to obtain a clear image of both legs. Knee varus/valgus was measured as the angle between the longitudinal axes of femur and tibia via the sterEOS software. (Version 1.6, EOS imaging, Paris, France). Anatomical reference points were identified on both the sagittal and coronal planes, and bony contours were adjusted according to the EOS guidelines. Between-day reliability was assessed in six participants and the result was excellent (ICC = 0.99, *p* < 0.001).

### Statistical analysis

Statistical analysis was conducted with R software (Version 4.02). For participants with bilateral symptoms, analyses were conducted on the more painful leg. All the variables were normally distributed as assessed by Shapiro-Wilk test. Correlations between VAS pain, KOOS pain, KAM, KFM, radiographic severity, knee varus angle and walking speed were assessed by two-tailed Pearson correlation coefficient. The relationship between pain intensity and KAM were estimated by path analysis with maximum likelihood estimation. There was one missing data in the KOOS pain subscale and it was handled by pairwise deletion in the analysis.

Path analysis was an extension of multiple regression analysis using correlational data to discover the strength of effect of a hypothesized system [30]. It had the advantage to estimate both direct and indirect effects between variables. Pain intensity, KAM and KFM were the endogenous variables whereas walking speed, OA radiographic severity and knee varus angle were the exogenous variables. The results of correlation between exogenous variables were not shown in the model. The goodness-of-fit criteria was assessed by Chi-square, Comparative Fit Index, Tucker-Lewis Index, Root Mean Square Error of Approximation and Standardized Root Mean Square Residual [31]. The model proposed in this study was a conceptual model of the relationships between knee joint loadings and pain intensity sourced from the literature.

Correlations between VAS pain, KOOS pain and KAM index were assessed by partial Pearson correlation coefficient test controlling for radiographic severity, knee varus angle and walking speed.

## Results

A total of 100 participants with knee OA were initially screened and 47 who satisfied the study criteria were recruited. Main reasons for exclusion were low back pain, recent knee injury or surgery and lateral knee OA. The Information of the included participants was shown in Table 1. Their mean age was 62.1 ± 6.0 years old and 78 % of the participants were females. Thirty participants (64 %) were categorized as mild knee OA. All except 4 participants had bilateral knee OA.

**Table 1:** Descriptive information

Characteristics	(*n* = 47)
Demographic information	
Age (years)	62.06 ± 6.01
BMI (kg/m^2^)	26.25 ± 3.56
Gender (female/male)	37/10
Mild/Moderate	30/17
Bilateral/Unilateral	43/4
Knee varus angle (°)	6.02 ± 5.27
Walking speed (m/s)	1.00 ± 0.17
Knee joint kinetics	
External knee adduction moment (Nm/kg)	0.50 ± 0.15
External knee flexion moment (Nm/kg)	0.47 ± 0.26
KAM index (%)	54.12 ± 17.41
Pain	
VAS	5.15 ± 1.76
KOOS	63.08 ± 14.02

### Relationships between self-perceived pain and joint loading

As shown in Table 2, VAS self-perceived pain intensity was positively related to KFM (*r* = 0.43, *p* = 0.003) and it had a negative association with KAM (*r*= -0.29, *p* = 0.05). The KAM had a negative relationship with KFM (*ρ*=-0.40, *p* = 0.01) and positive association with knee varus angle (*r* = 0.55, *p* < 0.001). There was less likely any relationship between KOOS pain intensity and KAM (*r*=-0.13, *p* = 0.40) or KFM (*r*=-0.01, *p* = 0.63). Nevertheless, greater pain intensity measured by VAS was associated with greater pain intensity measured by the KOOS (*r*=-0.39, *p* = 0.01).

**Table 2:** Correlation coefficient of variables in path analysis

	VAS Pain	KOOS pain	KAM	KFM	Severity	Walking speed	Knee varus angle
VAS pain^a^		-0.39**	-0.29*	0.43**	0.27	-0.02	0.04
KOOS pain^a^			-0.13	-0.07	0.01	0.01	-0.05
KAM				-0.40**	0.18	0.01	0.55**
KFM					0.01	0.15	-0.06
Severity						-0.33*	0.62
Walking speed							-0.31*

* *p* < 0.05, ** *p* < 0.01

^a^High VAS pain score indicates high pain intensity; low KOOS pain score indicates high pain intensity

### Path analysis

The model including VAS pain intensity was examined to be of good fit according to Chi-square test (*χ*^2^ = 4.89, df = 4, *p* = 0.30), Comparative Fit Index (CFI = 0.98) and Tucker Lewis Index (TLI = 0.93), which met the cut-off criterion [32]. The value for Standardized Root Mean Square Residual was 0.06 and that for Root Mean Square Error of Approximation was 0.07, and both were smaller than the threshold of 0.08. The effects of path model were shown in Table 3.

**Table 3:** Decomposition of effects from path analysis

Effect	Estimate	Standard error	Standard estimate	95% CI	*P* value
Model 1					
On VAS pain (*R*^2^ = 0.265)					
KAM	-2.50	1.61	-0.21	-5.66, 0.67	0.12
KFM	2.26	0.89	0.35	0.51, 4.01	0.01
Severity	1.09	0.45	0.31	0.20, 1.98	0.02
On KAM (*R*^2^ = 0.365)					
Varus angle	0.02	0.00	0.74	0.01, 0.03	< 0.01
Walking speed	0.13	0.11	0.16	-0.07, 0.34	0.21
Severity	-0.07	0.05	-0.23	-0.16, 0.02	0.12
On KFM (*R*^2^ = 0.192)					
Walking Speed	0.29	0.20	0.19	-0.10, 0.68	0.15
KAM	-0.71	0.24	-0.40	-1.17, -0.25	< 0.01
Model 2					
On KOOS pain (*R*^2^ = 0.036)					
KAM	-18.06	15.04	-0.19	-47.53, 11.41	0.23
KFM	-7.76	8.34	-0.15	-24.11, 8.59	0.35
Severity	1.04	4.25	0.04	-7.29, 9.38	0.81
On KAM (*R*^2^ = 0.358)					
Varus angle	0.02	0.00	0.74	0.01, 0.03	0.02
Walking speed	0.13	0.11	0.16	-0.08, 0.34	0.21
Severity	-0.07	0.05	-0.23	-0.16, 0.02	0.15
On KFM (*R*^2^ = 0.183)					
Walking Speed	0.28	0.20	0.19	-0.11, 0.67	0.16
KAM	-0.69	0.24	-0.39	-1.16, -0.23	< 0.01

**Fig. 1:**
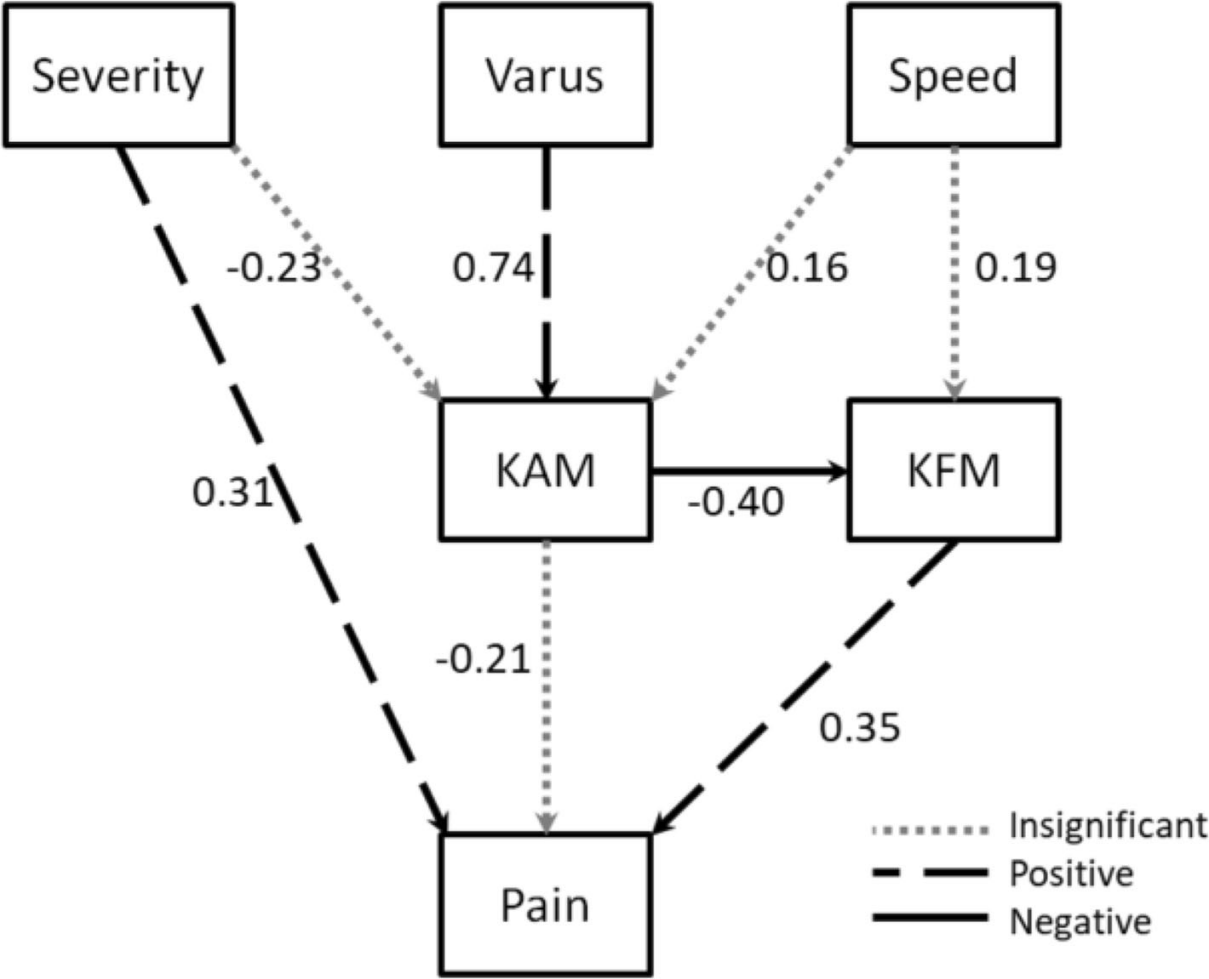
Diagram of path analysis. (Pain: intensity of pain using visual analogue scale. KAM: external knee adduction moment. KFM: external knee flexion moment. Varus : knee varus angle)

KAM was found to have an indirect effect on VAS pain intensity through KFM (see Table 4). No direct effect was found between KAM and VAS pain intensity. However, there existed a moderate negative relationship between KAM and KFM; the magnitude of KFM had a direct effect on VAS pain intensity. The effect of radiographic severity, knee varus angle and walking speed had been demonstrated in the path model (Table 3; Fig. 1).

**Table 4:** Effects of external knee adduction moments on intensity of pain measured by VAS and KOOS

	Estimate	Standard error	Standard estimate	95% CI	*P* value
VAS					
Direct	-2.50	1.61	-0.21	-5.66, 0.67	0.12
Indirect	-1.61	0.83	-0.14	-3.23, 0.02	0.05
Total	-4.10	0.16	-0.35	-7.21, -1.00	0.01
KOOS					
Direct	-18.06	15.04	-0.19	-47.53, 11.41	0.23
Indirect	5.38	6.07	0.08	-6.52, 17.27	0.38
Total	-12.69	14.09	-0.13	-40.30, 14.93	0.37

### Relationships between self-perceived pain and KAM index

**Fig. 2:**
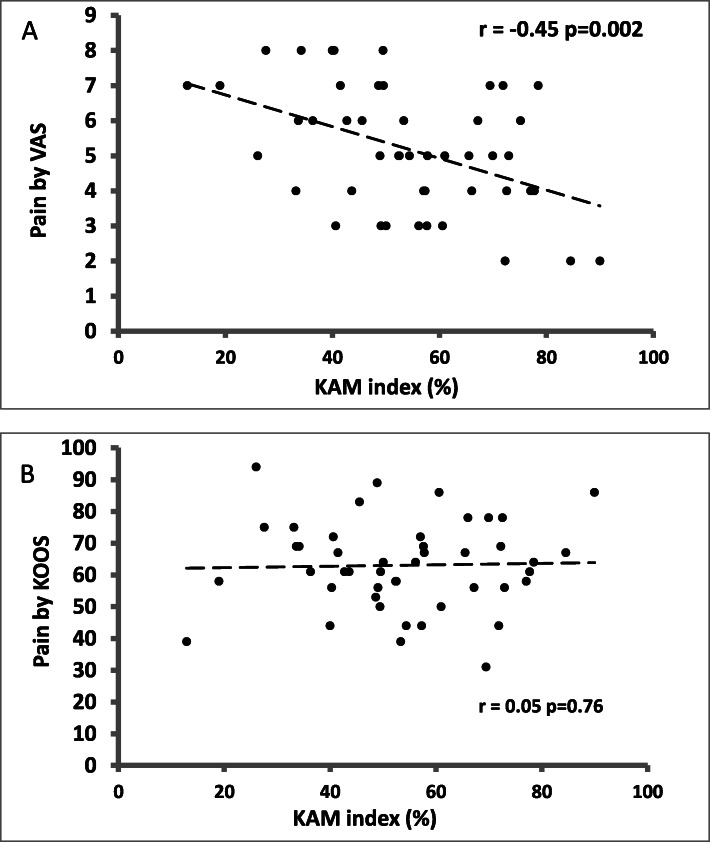
Scatter plots between pain intensity and KAM index controlling for disease severity, knee varus angle and walking speed. (VAS: visual analogue scale; KOOS: Knee Injury and Osteoarthritis Outcome Score)

Figure 2 showed the scatter plot between KAM index and pain intensity. There existed a negative association between the two variables (*r*=-0.45, *p* = 0.002) after controlling for radiographic severity, knee varus angle and walking speed. Hence, higher intensity of pain was associated with lower KAM index in participants with knee OA.

## Discussion

This study aimed to explore the relationships between pain intensity and knee joint loading in people with mild-to-moderate medial knee OA. Using path analysis, a more integrated conceptual framework was constructed. The findings suggested KAM had a negative effect on pain with KFM as its mediator. By using the KAM index, we also revealed a negative relationship with pain intensity in our participants.

Based on these findings, it is suggested that magnitude of KAM had a negative effect on pain intensity. The effect of KAM on pain was through the mediation of KFM. Negative relationship between VAS pain and KAM were reported in previous studies in people with mild radiographic severity of OA [15, 33]. The authors proposed such relationship could be an avoidance mechanism in response to pain provoking stimuli. Indeed, experimental pain induced in infrapatellar fat pad would significantly reduce the KAM and KFM during gait in healthy participants [34]. The authors further proposed the modulation on KAM and KFM was related to alteration in trunk motion, foot progression angle or muscle-coordination. However, OA-related pain is usually chronic and trigged by loading activity [9] and findings from healthy participants with experimentally induced pain might not be translated to participants with knee OA. Besides, during gait modification, reduction in KAM was associated with increase in KFM in participants with knee OA [34] which agreed with the present finding that moderate negative association was detected between KAM and KFM. In participants with knee OA, mechanical modification was proposed as the mechanism for the interchange between KAM and KFM.

We used the KAM index as an estimation of the percentage of KAM to the sum of KAM and KFM and found a negative relationship between this index and VAS pain intensity when adjusting for radiographic severity, knee varus angle and walking speed. This concurred with the results from path analysis that participants with lower share of mechanical loading on the frontal plane had less pain in walking. Since this was a cross-sectional observational study, a causal relationship could not be established. There could be other de-loading mechanisms adopted by the participants in response to painful stimuli via re-distribution of load between the frontal and sagittal planes, or higher load sharing on the frontal plane could have happened to minimize the walking pain. However, greater percentage of KAM to total external knee moment was linked with radiographic joint structural degeneration as measured by KL grading in 5 years [23]. Considering this point with the present findings, a higher KAM index would therefore be detrimental to the knee joint in the long run; its apparent association with lower pain was more likely to be associated with some other potential pain avoidance strategies, for instance, knee kinematics asymmetry between two limbs in mild-to-moderate knee OA [35]. However, this study cannot answer this question and further study is warranted to examine the mechanisms of how KAM index would modulate the pain intensity.

The KFM was found to be a mediator for the relationship between KAM and pain intensity in mild-to-moderate knee OA. In view KAM and KFM were antagonistic to one another, the present finding of a positive relationship between pain intensity and KFM echoed with the report of O’Connell et al. [33] that higher KFM was demonstrated in participants with moderate-to-severe pain than those with less pain [33]. Previous study had reported KFM was more sensitive to change in pain intensity than KAM over time in people with medial compartment OA [28]. The KFM was balanced with contraction of the quadriceps muscle which produced an internal knee extension moment [36]. The internal knee extension moment resulted from quadriceps contraction would induce a compressive force across the tibiofemoral joint [37] that might trigger pain.

Interestingly, the KOOS pain intensity and KAM were not likely interrelated either directly or indirectly. Likewise, pain intensity measured with the pain subscale of Western Ontario and McMaster Universities Osteoarthritis Index (WOMAC) was also not associated with the magnitude of KAM in radiographic medial compartment knee OA [38, 39]. A possible explanation might be because VAS was a unidimensional pain measurement tool focused on pain intensity localized at the knee joint whereas both KOOS and WOMAC were multidimensional measuring tools with more emphasis on disease progression and joint function. In fact, the KOOS pain subscale was an extension of WOMAC pain subscale, and they both measured pain intensity with Likert-type scales during several daily activities, including but not limited to level walking [40]. Apart from that, questions in KOOS and WOMAC pain subscales were not focused on a specific knee; thereby the pain on both knees for those with bilateral knee OA would influence the outcome. Though it is clarified that area of interest of is the knee in KOOS, low back pain and other musculoskeletal pain could also have an impact on the pain intensity scores when doing these physical activities [41]. Considering these holistic factors, KOOS and WOMAC pain subscales might have weaker relationship with magnitude of joint loading variables than knee pain in walking.

There were some limitations in this study that should be addressed. First, the sample size was considered small especially that of KL grade 3, which had restricted the model building. If the sample size was larger, a more comprehensive model could have been built. Second, the fact that severe knee OA participants were not included in the study has restricted the findings to be only applicable to patients with radiographic knee OA with KL grade less than 4. Finally, due to the cross-sectional nature of the study, measurements at a time point could not justify if any causal relationship did exist between pain intensity, KAM and other variables. Future interventional study will shed lights on how changes on KAM, KFM and pain intensity are interrelated.

## Conclusions

To conclude, in people with radiographic mild-to-moderate knee OA, KAM had a negative effect on self-perceived intensity of walking-related pain with KFM as its mediator. In these people, greater KAM index was associated with less walking pain. The distribution of knee joint loading from frontal to sagittal planes could be a pain avoidance strategy which has an application value for management of pain in people mild-to-moderate knee OA.

## Data Availability

The datasets used and/or analyzed during the current study are available from the corresponding author on reasonable request.

## Abbreviations

KAM: External knee adduction moment
KFM: External knee flexion moment
KL: Kellgren-Lawrence grading scale
KOOS: Knee Injury and Osteoarthritis Outcome Score
OA: Osteoarthritis
VAS: Visual analogy scale
WOMAC: Western Ontario and McMaster Universities Osteoarthritis Index
